# Physiotherapeutic interventions and physical activity for children in Northern Sweden with cerebral palsy: a register study from equity and gender perspectives

**DOI:** 10.1080/16549716.2017.1272236

**Published:** 2017-01-21

**Authors:** Frida Degerstedt, Maria Wiklund, Birgit Enberg

**Affiliations:** ^a^ Department of Community Medicine and Rehabilitation, Physiotherapy, Umeå University, Umeå, Sweden

**Keywords:** Disability, habilitation, physiotherapy, gender bias, CPUP registry

## Abstract

**Background**: Young people with disabilities, especially physical disabilities, report worse health than others. This may be because of the disability, lower levels of physical activity, and discrimination. For children with cerebral palsy, access to physiotherapy and physical activity is a crucial prerequisite for good health and function. To date, there is limited knowledge regarding potential gender bias and inequity in habilitation services.

**Objectives**: To map how physiotherapeutic interventions (PTI), physical leisure activity, and physical education are allocated for children with cerebral palsy regarding sex, age, level of gross motor function, and county council affiliation. This was done from a gender and equity perspective.

**Methods**: A register study using data from the Cerebral Palsy follow-Up Program (CPUP). Data included 313 children ≤18 years with cerebral palsy from the five northern counties in Sweden during 2013. Motor impairment of the children was classified according to the expanded and revised Gross Motor Function Classification System (GMFCS).

**Results**: In three county councils, boys received more physiotherapy interventions and received them more frequently than girls did. Differences between county councils were seen for frequency and reasons for physiotherapy interventions (p < 0.001). The physiotherapist was involved more often with children who had lower motor function and with children who had low physical leisure activity. Children with lower motor function level participated in physical leisure activity less often than children with less motor impairment (p < 0.001). Boys participated more frequently in physical education than did girls (p = 0.028).

**Conclusion**: Gender and county council affiliation affect the distribution of physiotherapy interventions for children with cerebral palsy, and there are associations between gender and physical activity. Thus, the intervention is not always determined by the needs of the child or the degree of impairment. A gender-bias is indicated. Further studies are needed to ensure fair interventions.

## Background

Globally, over 5% of children aged 0–14 years have a disability [1]. The same is true for the youngest children in Sweden [2]. At the age of 16 in Sweden, approximately 16% of children report having a disability that reduces physical, psychological, or intellectual functional ability. Twice as many disabled adolescents report being treated unfairly more than once, compared to non-disabled adolescents [3]. Children with disabilities are likely to have poorer health and less participation in society than others of the same age [1,4]. Reasons for the poor health may originate in the disability itself, or may be caused by external factors such as discrimination and low sense of control [4], as well as a low level of physical activity [4–6]. Among the disabled population, physically disabled persons report the most health problems [4]. Among children in Sweden, cerebral palsy is the most common cause of physical disability [7–9]. Approximately two of 1000 children aged 5–12 years are diagnosed with cerebral palsy [10], and boys are overrepresented by a factor of 1:1.4 in Sweden and western countries [11,12].

Cerebral palsy (CP) is an umbrella term that includes various motor impairments caused by damage to the immature brain [9,13]. This brain damage may also impact cognitive function, perception, and sensation [7–9]. Diagnosis of CP is usually made around the age of four; the motor impairment is classified according to the expanded and revised Gross Motor Function Classification System [14]. CP is a non-progressive disease that changes over time, e.g. consequences from spasticity and joint malformation on the growing body [15]. In Sweden, all children with diagnosed or presumed CP are offered participation in the Swedish national quality registry, the Cerebral Palsy Follow-Up Program (CPUP), which includes annual assessment of physical status, and mapping of interventions received within specialized habilitation services as well as physical activity [10].

### Swedish habilitation service

The Swedish habilitation service is an interdisciplinary public health service that specializes in disability among children, from birth to around 18 years of age. Habilitation is a complement to the regular public healthcare that all citizens are entitled to as part of the Swedish welfare model [16,17]. The habilitation service is often organized according to the World Health Organization (WHO) *International Classification of Functioning, Disability and Health* (ICF) [18]. The ICF includes body functions and structure, activity and participation, personal and environmental factors, and provides a common language regarding disability [18]. Physiotherapy interventions (PTI) within habilitation services aim to optimizing physical functions and abilities, as well as activity and participation for children. Physiotherapists cooperate with school personnel, relatives, or other professionals regarding physical activity and participation. They also work with other caregivers relating to orthotics, medication, surgery, etc [10,19]. Evidence supports strength training [20], constraint-induced therapy [21], activity-based intensive training [22], and physical activity through interactive gaming [19,23].

Body and movement in relation to health and illness are key within physiotherapy, and includes various forms of tailored physical activity. As such they are one important basis for habilitation interventions because they are crucial for participation in daily life and society [24,25]. Physical activity in leisure time, as well as physical education in school, is crucial to enhance function and maintain health [26,27]. Studies show that according to the Nordic recommendations of nutrition [26], children with CP do not have adequate physical activity, and their level of leisure activity decreases with age [6,24,28].

### Habilitation interventions on equal terms

The aim of the Swedish healthcare system is to offer good healthcare on equal terms [29]. The patient’s need of care is given preference in terms of not causing pain and enhancing autonomy and equality [29]. Nevertheless, differences in health assessments and clinical decisions that cannot be medically justified occur [19], and are associated with patient sex, gender, or other social characteristics [30–33]. Equity may be questioned when gender differences are overlooked leading to a generalized healthcare, as well as when improper beliefs about such differences affect the choice of examination or treatment [34]. The term *gender-bias* is used within medical gender research to identify and problematize the consequences of unconscious or conscious beliefs about sex or gender that can lead to inequity [34,35]. *Gender* is defined as the social and relational aspects of sex, rather than as a fixed state [34,36]. In terms of equal care and rehabilitation, gender often intersects with other social categories such as origin of birth, ethnicity, educational level, socioeconomic status, or home district/region of living [37]. In general, gender hierarchies and stereotypes leads to limitations in the participation and potential of the individual [38,39].

Lauruschkus et al. show that children with more severe motor impairment due to CP receive more physiotherapy interventions compared to those with lesser motor impairment [6]. Their study, based on CPUP data, show no significant differences between boys and girls with regard to physiotherapy interventions, but the type of intervention and presence of the PT was not specified. For children with CP, access to physiotherapy and physical activity is a crucial prerequisite for good health and function, as well as participation in society [25,40].

Children with disabilities are dependent on professionals within healthcare and in their environment. This makes it even more important to offer needed interventions on equal terms. To our knowledge, there is limited research regarding gender, gender bias, and other potential sources of inequity in habilitation and physiotherapy for children with disabilities such as CP. The aim of this study was to map how physiotherapeutic interventions (PTI), physical leisure activity, and physical education are allocated for children with cerebral palsy regarding sex, age, level of gross motor function, and county council affiliation. This was done from a gender and equity perspective.

## Methods

### Participants and data collection

This is an observational registry study with a cross-sectional design. Data from the Swedish National Quality Registry (CPUP), were compiled and provided by a registry keeper. Data were obtained through a coded file, after a September 2014 registry approval. The study included all children with CP in the age range of 0–18 years who were born between 1995 and 2013 and participated in CPUP the same year. The children were living in one of the five northernmost county councils in Sweden (Norrbotten, Västerbotten, Västernorrland, Jämtland, or Gävleborg) in 2013, and they participated in the CPUP the same year. Older children may be missing from this study because of a late connection to the registry by some county councils. In total, 313 children were included. No children under the age of one participated in the registry during 2013. At the age of 18, the children usually leave habilitation services.

### The cerebral palsy follow-up program

The Swedish national quality registry, the Cerebral Palsy Follow-Up Program (CPUP), started in 1994, in southern Sweden [10]. CPUP began as a cooperative project between pediatric orthopedics and the habilitation service to prevent hip dislocation and contractures among children with CP. The registry was also intended to spread knowledge about CP and thereby facilitate communication between different professionals. Participation in the registry is offered to all children who are thought to have CP even if the diagnosis is not stated. This is expected to capture at least 95% of all children between the ages of 0–18 years with known CP. The child’s local physiotherapist, occupational therapist, and pediatrician collect the CPUP registry data. The physiotherapist and occupational therapist usually make the physical and functional status assessments, including gross motor function according to the Gross Motor Function Classification System – Expanded and Revised (GMFCS-E&R). GMFCS-E&R has good content validity [14], good test-retest and inter-rater reliability, and validity for prediction of gross motor function. This means that the child usually stays at the same level while maturing [41]. GMFCS-E&R is divided in groups that describe each level, as roughly detailed in Table 1, and the factors that distinguish between them. GMFCS-E&R is supposed to mirror how the child usually chooses to move at home or in the community, rather than his/her capability of movement [14]. The assessments are made twice a year until the sixth birthday, and annually thereafter. The exceptions are children with very mild impairment who are followed less often according to CPUP recommendations [10]. Other collected data are related to the child’s or child’s relative’s estimation of the child’s situation during the period since the last CPUP assessment. The pediatrician makes one assessment after the fourth birthday, and then whenever there is new information about test results related to additional diagnoses or visual or cognitive impairment. Apart from surgical data, which is partly collected by the pediatrician, this study only includes data from the physiotherapy assessment [10].

**Table 1.** Description of gross motor function levels and merged groupings according to the Gross Motor Function Classification System-Expanded and Revised (GMFCS-E&R) used in this study.

Level GMFCS-E&R according to original scale		Merged levels
I	Walks without limitations	GMFCS-A
II	Walks with limitations	
III	Walks using a handheld mobility device, uses manual wheel chair independently	GMFCS-B
IV	Self-mobility with limitations. May use powered mobility	GMFCS-C
V	Transported in a manual wheelchair	

In the present study, 73 children received two assessments during the year; twenty of the children were older than age 6 years. Only the first assessment was used in this study. Collected data included sex, age, GMFCS-E&R, and county council affiliation. Further, information about receipt of botulinum toxin (botox), medication for spasticity, surgery, and the use of orthotics on lower extremity were collected as background variables since they are presumed likely to affect PTIs and the opportunities for physical activity.

For this study participants were grouped by age: 0–5 yr, 6–11 yr, and 12–18 yr. To facilitate analyses, the levels were merged into three instead of five levels (Table 1). Levels I and II were merged into GMFCS A, Level III is a separate group, GMFCS B, and levels IV and V were merged into GMFCS C.

Physiotherapeutic interventions (PTIs) were defined as guidance and interventions, except CPUP assessments for the purpose of examination, prevention or treatment of dysfunction that restrict or may restrict the child’s ability to move. The interventions could have been performed individually or in groups. The main focus was on the interventions received rather than who performed them. Data does not report duration of each intervention. Data on PTIs since the last assessment (yes/no), if yes, how often (< once a month, 1–3 times/month, once/twice a week, 3–5 times/week, >5 times/week) were included. We also included whether the physiotherapist was present (same frequency alternatives as above). The alternatives for PTIs were dichotomized into ‘often’ (≥ 2 times/week) and ‘rarely’ (≤1–3 times/month). When presence of the physiotherapist during the intervention was evaluated, the groups were altered so that ‘often’ included 1–3 times/month and ‘rarely’ included only those with a physiotherapist present less than once a month. This is because the role of physiotherapist is often delegated to people in the child’s vicinity and is not performed by a physiotherapist.

Data included information on participation in a period of intensive training (yes/no), and the intention of the PTI: muscle force, tone or endurance; joint mobility; postural ability; aerobic capacity; body awareness; respiration; pain (yes/no). Engagement in physical leisure activity and physical education/activity in school were asked in separate questions (yes/no). If the answer was yes, then this was categorized by frequency (<1 time/week, 1–2 times/week, 3–5 times/week). These answers were dichotomized into ‘often’ (≥1–2 times/week) and ‘rarely’ (< once a week). By physical education we refer to physical activity as a school subject. Children in the age group of 0–5 yr, who are not in school, were excluded from the physical education analyses. Type of physical leisure activity was reported (swimming, horseback riding, soccer, dancing, strength training, gymnastics, skiing, skating, basketball, boccia, archery, sledge hockey, and other). Soccer, basketball, table tennis, and floor ball were merged into ‘ball sports’ and reported under ‘other’. Bicycle and martial arts were given separate variables in the category ‘other’. Different activities were compiled into one variable to indicate the number of different activities for each participant (ie, no activity, one activity, two or more activities) without regard to the activity.

### Statistical analysis

Data analyses were done using IBM SPSS Statistics v 23. Chi-squared test with Monte Carlo analyses were used to compare groups. Univarate and multivariate logistic regressions analyses were applied to test associations between receiving physiotherapeutic interventions, receiving physiotherapeutic interventions often and the independent variables sex and county council. Multivariate logistic regressions were made regarding the independent variables sex and country council which were considered more essential regarding the equity and gender perspective. Univarate and multivariate logistic regressions analyses were applied to test associations between Physioterapist present ‘often’ and intensive training and independent variables Sex, age group, county council and GMFCS-group. Odds ratios (OR) and 95% confidence intervals (CI) were calculated.

### Ethics

The National Registry CPUP received approval from the ethics committee for studies that only contain data from the database. That approval is consistent with this study [10], and additional approval was not sought or needed. The current study followed the Declaration of Helsinki [42] and ethical principles and operational guidelines for good clinical practice in paediatric research [43], and is reported so that individual children and physiotherapists cannot be identified.

## Results

### Demographic and clinical characteristics

Among the 313 study participants, 141 (45%) were girls. Nearly half the group (49%) was aged 6–11 years, and least number of participants (17%) were in the 11–18 year age group (Table 2). Most participants were classified in GMFCS-A (58%) and the fewest were in GMFCS-B (9%). In two cases, information about gross motor function level was missing however remaining data regarding those participants was not excluded. Majority of children belonged to GMFCS-group A. Nearly half of the children were in age group 6–11. (Tables 2–3). Eighty-six (30%) of 291 (93%) participants received botox in the lower extremities. Forty-six (16%) of 290 children reported taking spasticity-reducing medication, and 38 (12%) had some type of surgery. One hundred and sixty-three (55%) of 296 (96%) reported wearing orthotics for lower extremities.

**Table 2.** Distribution of sex, age, GMFCS group, county council affiliation, receipt of botox treatment, medication for spasticity, surgery or wears orthotics, by sex.

Variable		Girls n = 141	(%^b^) (45)	Boys n = 172	(%^b^) (55)	Total N = 313
Age (years)	0–5	46	(44)	59	(56)	105
(n = 313^a^)	6–11	68	(44)	86	(56)	154
	12–18	27	(50)	27	(50)	54
GMFCS-E&R	GMFCS-A	85	(47)	96	(53)	181
(n = 311)	GMFCS-B	10	(36)	18	(64)	28
	GMFCS-C	45	(44)	57	(56)	102
County Council	Norrbotten	14	(38)	23	(62)	37
(n = 313)	Västerbotten	28	(35)	52	(65)	80
	Västernorrland	35	(49)	37	(51)	72
	Jämtland	23	(54)	20	(47)	43
	Gävleborg	41	(51)	40	(49)	81
Botox (n = 291)	yes	35	(41)	51	(59)	86
Spast. med^c^. (n = 290)	yes	22	(48)	24	(52)	46
Surgery (n = 313)	yes	20	(53)	18	(47)	38
Orthotics^d^ (n = 296)	yes	75	(46)	88	(54)	163
	training	25	(46)	29	(54)	54
	balance	52	(45)	64	(55)	116
	gait	18	(53)	16	(47)	34

^a^n varies depending on number of participants reporting on each variable.

^b^Percentage refers to boys and girls in each row.

^c^Spasticity reducing medication.

^d^Participants may have more than one reason for use of the orthotics.

**Table 3.** Distribution of GMFCS-group in percent in each age group. Chi2 (p = 0.031).

Age group	n	GMFCS-A	GMFCS-B	GMFCS-C
0–5	105	61 %	7 %	27 %
6–11	154	62 %	10 %	27 %
12–18	54	42 %	9 %	50 %
Total (n)	313	181	28	102

### Physiotherapeutic interventions

Two hundred and forty-eight (79%) of 293 (94%) participants reported PTI since their last assessment. More of the boys (89%) than girls (79%) had PTI in the previous assessment period (p = 0.016) (Tables 4–5).

**Table 4.** Rate of participants (%) who responded yes in variables concerning physiotherapy interventions and physical activity by sex, age group and GMFCS group.

Intervention/activity	Sex			Age-group				GMFCS-group			
(n)	F %	M %	p-value	0–5 %	6–11 %	12–18 %	p-value	A %	B %	C %	p-value
											
PTI yes (248)	89	79	0.016^x^	88	82	87	0.42	75	100	96	<0.001^x^
PTI often (149)	54	68	0.025^x^	65	62	58	0.72	58	67	66	0.45
PT^a^ present often (93)	39	38	0.95	46	31	46	0.068	29	48	50	0.005^x^
Intensive training (47)	17	17	0.98	11	19	21	0.21	17	30	13	0.11
Factors aimed to improve by PTI:											
Strength (197)	68	73	0.34	75	67	75	0.33	62	96	78	<0.001^x^
Tone (145)	49	57	0.20	57	49	58	0.38	42	80	66	<0.001^x^
Joint mobility (242)	85	88	0.51	88	86	85	0.85	80	96	95	0.002^x^
Postural ability (200)	71	74	0.50	81	67	74	0.57	66	89	81	0.006^x^
Aerobic capacity (116)	48	39	0.15	36	43	54	0.12	42	58	40	0.25
Body awareness (124)	43	49	0.29	54	41	47	0.14	38	46	61	0.002^x^
Respiration (51)	22	17	0.31	17	14	36	0.003^x^	9	13	38	<0.001^x^
Pain (37)	16	12	0.35	9	12	29	0.003^x^	12	13	19	0.31
											
Leisure act YES (177)	57	56	0.77	45	63	61	0.011^x^	65	57	42	0.001^x^
Leisure act Often (78)	79	74	0.52	66	80	79	0.27	81	71	66	0.24
Phys edu^b^ Often (198)	88	96	0.028^x^		94	93	0.20	93	96	87	0.29

^x^P < 0.05 are considered significant.

^a^Physiotherapist.

^b^Physical education.

**Table 5.** Rate of participants (%) who responded yes in variables concerning physiotherapy interventions and physical activity by county council.

	County council					
Intervention/activity (n)	Norrbotten %	Västerbotten %	Gävleborg %	Jämtland %	Västernorrland %	p-value
Received PTI (248)	92	95	70	88	82	<0.001^x^
Received PTI often (149)	90	84	15	68	56	<0.001^x^
PT^a^ present often (93)	44	37	32	29	50	0.23
intensive training (47)	9	23	17	21	11	0.19
Factors aimed to improve by PTI:						
Strength (197)	95	74	44	71	77	<0.001^x^
Tone (145)	92	47	40	33	62	<0.001^x^
Joint mobility (242)	92	95	68	90	86	<0.001^x^
Postural ability (200)	95	87	49	68	66	<0.001^x^
Aerobic capacity (116)	68	41	26	21	59	<0.001^x^
Body awareness (124)	81	65	9	32	45	<0.001^x^
Respiration (51)	41	20	0	16	24	<0.001^x^
Pain (37)	35	11	4	5	20	<0.001^x^
Leisure activity YES (177)	60	65	47	63	53	0.16
Leisure act. Often (78)	75	68	72	79	89	0.39
Phys edu^b^ often (198)	96	92	87	90	96	0.44

^x^p-values <0.05 is considered significant.

^a^Physiotherapist.

^b^Physical education.

Univariate logistic regression showed significant associations between receiving PTI and sex. Boys had a higher odds of participation compared to girls (OR 2.2, 95 % CI 1.1–4.2). PTI and county council affiliation were also associated. With Gävleborg as the reference county, the odds of receiving PTI were higher in Västerbotten (OR 8.3, 95 % CI 2.7–25.7), Norrbotten (OR 4.8, 95 % CI 1.32–17.45), and Jämtland (OR 3.2, 95 % CI 1.1–9.4) (Table 6).

**Table 6.** Odds ratios (OR) and 95 % confidence interval (95 % CI) from univariate and multivariate logistic regression for associations between receiving physiotherapy interventions, county council affiliation and sex.

Variable	Participants (n = 293)	OR Univariate	CI (95 %)	OR Multivariate	CI (95 %)
Sex					
Girls	134	1		1	
Boys	159	2.2	1.2–4.2	1.9	1.0–3.8
County Council					
Gävleborg	69	1		1	
Norrbotten	36	4.8	1.3–17.5	4.5	1.2–16.3
Västerbotten	80	8.3	2.7–25.7	7.5	2.4–23.5
Västernorrland	66	2.0	0.9–4.4	1.9	0.9–4.4
Jämtland	42	3.2	1.1–9.4	3.3	1.1–9.7

Within Gävleborg County Council, 81% of boys and 60% of girls received PTI (p = 0.05). In Gävleborg, receipt of PTI by gross motor function was 59% in GMFCS-A, 100% in GMFCS-B, and 93% in GMFCS-C (p = 0.003). In Västernorrland, receipt of PTI was 68% in GMFCS-A, 100% in GMFCS-B, and 96% in GMFCS-C. No statistically significant differences in PTI were seen between sex, age group, or GMFCS-group within other county councils.

Two hundred and forty participants (77%) reported their PTI frequency. Among those who received the interventions ‘often’ was reported by 54% of girls and 68% of boys (p = 0.025) (Table 4).

Univariate logistic regression found associations between ‘PTI often’ and sex. The frequency of ‘PTI often’ was higher for boys than girls (OR 1.86, 95CI 1.08–3.10). Compared to Västernorrland, the odds of ‘PTI often’ were higher in Gävleborg (OR 7.13, 95 % CI 2.71–18.62), and in Norrbotten (OR 52.48, 95 % CI 12.84–214.67) (Table 7).

**Table 7.** Odds ratios (OR) and 95% confidence interval (95% CI) from univariate and multivariate logistic regression analyses for associations between ‘physiotherapy interventions often’, county council affiliation and sex.

Variable	Participants (n = 240)	OR Univariate	CI (95 %)	OR Multivariate	CI (95 %)
Sex					
Girls	102	1		1	
Boys	138	1.9	1.1–3.1	1.8	0.9–3.3
County Council					
Västernorrland	53	1		1	
Norrbotten	31	52.5	12.8–214.7	52.5	12.7–216.1
Västerbotten	75	29.5	11.2–78.1	28.7	10.8–76.3
Jämtland	38	12.2	4.4–33.7	12.8	4.6–35.7
Gävleborg	43	7.1	2.7–18.6	7.1	2.7–18.8

Significant differences between boys and girls receiving ‘PTI often’ were seen in two county councils. In Norrbotten, 75% of girls and all boys received ‘PTI often’ (p = 0.02). In Jämtland, 53% of girls and 84% of boys (p < 0.04) did so. In Västerbotten, receipt of ‘PTI often’ by GMFCS group was 74% for GMFCS-A, 100% for GMFCS-B, and 93% for GMFCS-C (p = 0.027). No other differences were seen for ‘PTI often’ between sex, age group, or and GMFCS-group within the county councils.

Among the 240 participants who indicated whether a physiotherapist was present at the PTI, no significant differences were seen between girls and boys, or county council affiliation. A physiotherapist was present at interventions for 29% of the participants in GMFCS-A, 49% in GMFCS-B, and 50% in GMFCS-C (p = 0.005). The odds of having a physiotherapist present ‘often’ was 2.4 times higher in the GMFCS-C group compared to GMFCS-A (OR = 2.4, 95 % CI 1.4–4.3) after controlling for sex, age group, and county council affiliation. The comparison to the GMFCS-B group was not statistically significant.

Among participants noting the reason that they received PTI, no significant differences were seen between girls and boys (Table 4). A larger share of participants in age group 12–18 had interventions to facilitate respiration and ease pain compared to younger age groups (p = 0.003). More children in GMFCS-B had interventions to facilitate muscle strength (p < 0.001), muscle tone (p < 0.001), and postural ability (p < 0.006) compared to GMFCS-A & -C. Regarding body awareness and joint mobility there were more participants in GMFCS-C compared to – A and – B (p = 0.002) which also was the case regarding interventions promoting respiration (p < 0.001). Between county councils there were statistically significant differences regarding purpose of PTI (p < 0.001) (Table 5).

Two hundred and eighty-one (90%) participants responded to if they had participated in a period of intensive training since their last assessment (Table 4). Univariate logistic regression showed that participants in GMFCS-B had 2.91 times higher odds to participate in intensive training compared GMFCS-C (OR = 2.91, 95 % CI 1.05–8.11). No significant associations were seen compared to GMFCS-A.

### Physical leisure activity and physical education

One hundred and seventy-seven (57%) participants stated that they participated in physical leisure activities (Tables 4–5). Among those 130 (73%) responded to how often that happened. No statistically significant difference was observed regarding sex and county council affiliation. In age group 0–5 45% of the children participated in leisure activities compared to 63% in age group 6–11 and 61% in age group 12–18 (p = 0.011). Sixty-five percent of the children in GMFCS-A had leisure activities compared to 57% in GMFCS-B and 42% in GMFCS-C (p < 0.001).

One hundred and sixty nine (54%) did not state any specific physical leisure activity that was performed. The remaining 144 have indicated 1–8 different activities relatively equally between girls and boys and between GMFCS-groups. Among participants in age group 0–5 21% noted one leisure activity and 11% more than one. Corresponding figures in age group 6–11 were 34% and 19% and for age group 12–18 33% and 20% (p = 0.018). As for the distribution of leisure activity in different county councils 77% of participants in Gävleborg did not report a specific leisure activity to participate in, 22% reported one activity and 1% reported several activities. Regarding Västernorrland corresponding figures were 58%, 29% and 13%, Jämtland 44%, 23% and 33%, Norrbotten 41%, 41% and 19% and in Västerbotten 39%, 35% and 26%. (p = 0.001).

Frequency of participation in physical education was reported by 215 (69%) participants. The majority (92%) participated at least once a week, and this included 88% of girls and 96% of boys (p = 0.028). The three most common activities for girls were swimming, skiing/skating and riding and for boys swimming, ball sports and skiing/skating. For No differences were seen between age groups, GMFCS-groups, or county councils.

In relation to whether or how often they engaged in leisure activities or physical education, there were no significant differences between groups for extent of PTIs or intensive training periods. Among participants who had leisure activity, 31% had a physiotherapist present during PTI compared to 48% of those who did not participate (p < 0.01).

### Other clinical variables

No statistically significant differences were seen between sex and receiving botox, spasticity-reducing medication, surgery, or orthotics. There were no differences between county council affiliation and surgery or orthotics. However, differences existed between the five county councils for botox. More children than expected received botox in Norrbotten, and fewer than expected in Västerbotten (p < 0.001). Medication to reduce spasticity was received more often than expected by children in Norrbotten and Västernorrland, and fewer than expected did so in the other three county councils (p = 0.001).

## Discussion

We aimed to map how physiotherapeutic interventions, physical leisure activity, and physical education are allocated for children with CP from an equity and gender perspective. The odds of receiving PTI, and of receiving it often, was higher for boys than girls. This indicates a gender-bias in access to habilitation services. This unequal distribution of habilitation resources is based on other (social) factors, and not just degree of impairment and individual needs. The gender differences found here are in contrast to a study from southern Sweden in which no gender differences were found for PTIs or physical education [6]. This might be because of small groups since that study did not merge alternative answers to form larger, more groups. However, different results from different studies, and from different county councils within the current study, reinforce the importance of further investigation into inequities between county council affiliations since the current study found significant differences in frequency and intensity of PTIs. We did not consider whether the children live in urban or rural areas, or distance from the habilitation services, so it is not possible to determine whether the differences are because of geographical distances or varied economic resources within the county councils or because of a more systematic bias.

Our focus was on the physiotherapeutic intervention that the child received, rather than on who performed the intervention. Interventions are commonly tailored and taught by the physiotherapist but performed by parents or persons close to the child. In the current study, a physiotherapist was more often present during PTIs for children with GMFCS-B and GMFCS-C levels. At a first glance, this may be interpreted as inequity between groups. However, these differences more likely reflect an increasing need for guidance by a physiotherapist when more severe motor impairment is present. Such oversight may be needed to obtain a satisfactory level of activity and participation, and this would not reflect an inequity. More of the participants in the GMFCS-C group were aged 12–18 years old; this may explain the increased presence of a physiotherapist in this age-group. Physiotherapists were also more frequently in attendance for the 0–5 year old age group; this is probably because there are more developmental changes for young children and these call for more frequent support for the children and their families.

The majority of children with CP are not sufficiently physically active according to Nordic recommendations for good health [26,28,44]. This may be why a physiotherapist is more often present at PTIs for children who are not engaged in leisure activity. If this is the case, our results may indicate a (fair) distribution of PT resources that are based on need, and a goal of physical activity promotion and enhancement among groups who need this intervention. Previous mappings of physical activity for children with CP show that more severe motor impairment is associated to less leisure activity [6]. This is in accord with our results. These studies also found that leisure activity decreased with age [6,28], a finding that corresponds with our results and a Swedish national study of children’s physical activity [44]. This lessening of activity with age is a problem since health problems deriving from lifestyle factors such as low level of physical activity are usually founded in childhood but appear later in life [44].

Since all treatment limits a child’s freedom to some extent, and takes time and energy from other activities, may mean that more treatment is not always desirable. Nevertheless, PTIs can be crucial for optimized development of the children [19]. This clarifies the value of a carefully balanced distribution of habilitation resources that is based on individual needs rather than gender, county council affiliation, or other factors.

Our results regarding physical leisure activity and physical education in relation to sex/gender are interesting. Brunton et al. point to a tendency for girls with CP to be less physically active than boys [28]. The same gendered pattern was shown in the general population of Swedish children [44], but was not confirmed by the current study. Like national studies, our results show more frequent participation in physical education among boys than girls [44]. One possible reason for this is gendered constructs related to physical activity, e.g. how girls and boys are described within the world of sports. This includes the (different) expectations set for girls and boys [26]. For example, in educational books for young athletic leaders, the boys are described as ‘naturally athletic’ and having positive development in puberty. In contrast, a girl’s puberty is often described as a problem in relation to sports, and focus is on the connection between femininity and fertility [45]. These differences in gender perceptions related to physical activities risk creation of different gendered expectations for boys and girls, both from themselves and the environment [45]. Other studies have not found gender differences in physical education [6,28]. As physical education may be the first and only contact with physical activity, and thereby connected to health, further investigation as to why girls participate less in physical activity is important.

No associations were seen between GMFCS-group and physical education. This was a positive finding since many children with CP are in specialized schools where physical education is expected to be customized to each child. This contrasts to the findings of Lauruschkus et al., who reported less participation for children in GMFCS-E&R level V [6]. Merging of GMFCS levels into groups may have hidden differences in the current study. A larger proportion of participants in GMFCS-C did not respond to the questions about physical education, and this makes our findings less certain.

### Study limitations and strengths

Children in the 6–11 age group are overrepresented in the CPUP registry. Fewer participants in the 12–18 age group is likely because they were not all in the age groups initially included in CPUP by each county council. For example, Västerbotten joined the registry in 2003, and included children born in or after 1999. Some older children were assessed with CPUP guidelines, but not reported to the registry. In 2007, all county councils participated in the registry. Norrbotten was among the last to join, and Gävleborg and Jämtland joined in 2006. The registry will be more complete with each progressing year, and further studies will include more participants and thereby have more accurate results. In the 0–5 age group, some children with mild CP are likely to be missing because they do not have a detected diagnosis [10].

When two assessments were done during 2013, the current study only includes the first. Among the 73 children who had two assessments, there were no gender differences. Twenty were older than 6 years of age, and therefore received an additional assessment that was beyond CPUP guidelines. Among those 20 children, fourteen were in the same county council. Further investigation from a national perspective of the associations of factors with PTIs would be interesting.

Factors that were not obtained by or from the registry could affect the results. For example, which parent accompanies a child to the habilitation ward, distance to the habilitation ward, socioeconomic status, and ethnicity might all be important factors that we did not assess. Additional data such as the different dominating symptoms [6,8,12], place of birth, place of residence, additional diagnoses, and data on activities and participation should be included in future studies. Surveys or interviews that provide the perspective of the children with CP, their parents, and physiotherapists in the habilitation wards that focus on PTIs, physical activity, and habilitation on equal terms, would enrich our knowledge.

## Conclusions

Gender and county council affiliation affect the distribution of PTIs for children with CP in the north of Sweden. Physical leisure activity is influenced by age and GMFCS-group, and physical education is influenced by gender. Differences regarding botox and spasticity reducing medications were seen in different county councils, but there were no differences between sexes. Thus, our results point out that the distribution of, access to, and choice of PTIs is not always determined by the needs of the child or the degree of impairment. From a perspective of equity and gender, children with CP need to have the opportunity to receive interventions based on individual needs rather than based on social aspects such as sex or county council/country region. A broader national mapping of differences between groups and associations would be valuable, and should be complemented by in-depth analyses of the causes of these differences. For example, qualitative interview studies are needed with habilitation professionals (such as physiotherapists), children, and their families. Further studies within national and global contexts are needed to ensure the quality of care and interventions. Such needed studies include access to, and distribution of, physical activity and physical education in the habilitation services to ensure that children with CP receive fair treatment that is based on individual needs.

## Paper context

Cerebral palsy is the most common cause of physical disability in children. These children have poorer health and less participation in society than others, e.g. because of the disability, lower levels of physical activity, and discrimination. From equity and gender perspectives, we map how physiotherapeutic interventions, physical leisure activity and physical education are allocated for children in northern Sweden with cerebral palsy. Our main results indicate a gender-bias in access to habilitation service.
